# A unique radioprotective effect of resolvin E1 reduces irradiation-induced damage to the inner ear by inhibiting the inflammatory response

**DOI:** 10.1186/s13014-020-01662-9

**Published:** 2020-09-25

**Authors:** Jie Zhang, Anting Xu, Tingting Niu, Chengcheng Liu, Yongju Zhang, Tao Li, Jihua Wang, Yongjing Wang, Dianshui Sun

**Affiliations:** 1 Department of Otolaryngology & NHC Key Laboratory of Otorhinolaryngology, the Second Hospital of Shandong University, Jinan, 250033 Shandong Province China; 2 Department of Medical Technology, the Jinan Vocational College of Nursing, Jinan, 250102 Shandong Province China; 3 Cancer Center, the Second Hospital of Shandong University, No.247 Beiyuan Road, Jinan, 250033 Shandong Province China; 4 Department of Hematology, the Second Hospital of Shandong University, Jinan, 250033 Shandong Province China

**Keywords:** Inner ear, Resolvin E1, Inflammatory response, Proinflammatory cytokine, Radioprotective effect

## Abstract

**Background:**

In addition to the direct effects of irradiation, the induced inflammatory response may play an important role in the damage to the inner ear caused by radiotherapy for the treatment of head and neck cancers. Resolvin E1 (RvE1) has anti-inflammatory activity, acting by reducing neutrophil infiltration and proinflammatory cytokine expression. Therefore, in this study we sought to confirm whether the inflammation induced by irradiation was involved in damage to the inner ear after radiotherapy and to investigate the protective effect and underlying mechanism of RvE1 using mouse models.

**Methods:**

A dose of RvE1 was delivered by intraperitoneal injection to mice before irradiation. Changes in the auditory brainstem response (ABR), relative balance ability, inner ear morphology and the expression levels of inflammatory factors in the inner ear were analyzed on days 7 and 14 after irradiation and compared among different experimental groups.

**Results:**

Changes of ABR and relative balance ability showed the inner functions of experimental mice presented severe damage after irradiation, but the damage was significantly alleviated after RvE1 pretreatment compared to irradiation alone. Morphological analysis of the inner ear showed severe damage to the cochlea and vestibule after irradiation. In contrast, damage to the cochlea and vestibule was significantly reduced in the RvE1-pretreated group compared to that in the irradiation alone group. Along with these functional and morphological changes, the mRNA expression level of anti-inflammatory factors interleukin-2 was significantly increased, while those of proinflammatory factors interleukin-6 and tumor necrosis factor-α were significantly decreased in the inner ear of mice after RvE1 pretreatment compared to irradiation alone.

**Conclusions:**

We believe that inflammation induced by irradiation is involved in the damage to the inner ear caused by radiotherapy, and that RvE1 reduces the damage caused by irradiation to the inner ear by regulating the induced inflammatory response.

## Introduction

Head and neck cancer is one of the most common malignancies. According to epidemiological surveys, the annual incidence of head and neck cancer in China is 15.22/100,000, which accounts for 4.45% of all cases of malignant tumors. Radiotherapy is an important treatment for head and neck cancer. Although radiotherapy can achieve satisfactory therapeutic effects in such cases, damage to adjacent tissues and organs is inevitable. The inner ear is one organ commonly affected by irradiation, resulting in sensorineural hearing loss (SNHL) and vestibular dysfunction [1–4]. For the majority of patients with advanced head and neck cancer, long-term survival can be achieved after multidisciplinary treatments including radiotherapy [5, 6]. Thus, irradiation-induced SNHL and vestibular dysfunction are inevitable consequences affecting the life of these cancer survivors [7], and protecting the inner ear from damage following irradiation would improve the quality of life of these cancer survivors. SNHL and vestibular dysfunction after irradiation are caused by damage to the inner and outer hair cells, spiral ganglion and vestibular hair cells. Direct effects of the irradiation dose on the inner ear are not solely responsible for the damage to the cochlea and vestibule [8, 9]. Some studies have reported that the inflammatory response induced by irradiation can cause apoptosis of hair cells. Our previous clinical study found an abnormal increase of interleukin (IL)-6 in the peripheral blood of patients with irradiation-induced inner ear damage [9], and another study found that a large number of inflammatory cells appeared in the vestibular tissue of irradiated guinea pigs [10]. These studies imply that the inflammation induced by irradiation may play an important role in damage to the inner ear, but there is a lack of complete experimental evidence in support of this hypothesis.

Resolvin E1 (RvE1) is biosynthesized from the omega-3 polyunsaturated fatty acid eicosapentaenoic acid (EPA), and shows remarkable potency in promoting resolution of inflammation and helping to prevent progression of an acute inflammatory response into chronic inflammation [11, 12]. Anti-inflammatory effects of RvE1 have been previously studied and reported in many inflammation-related diseases and in several animal models of inflammation such as diabetic nephropathy, periodontitis, asthma, sepsis and acute respiratory distress syndrome [13–17]. The mechanisms of RvE1 in counteracting inflammation and promoting its resolution include suppression of procytokine expression/release, inhibition of leukocyte recruitment resulting in a reduction in the amount of local and circulatory cytokines that attenuate the inflammatory responses, and induction of macrophage phagocytosis of apoptotic polymorphonuclear neutrophils [15, 18].

Radiotherapy induces a local inflammatory response in irradiated tissues including leukocyte recruitment and procytokine expression/release [19]. It was unclear whether the inflammatory response induced by irradiation was involved in the damage to the inner ear after radiotherapy, and whether RvE1 had a radioprotective effect on the inner ear by inhibiting the inflammatory response. Therefore, in this study a mouse experiment was designed to investigate these problems and the potential mechanism of RvE1 in protecting against irradiation-induced damage to the inner ear.

## Materials and methods

### Animals 

One-month-old C57BL/6 female mice weighing 25–27 g were purchased from the Experimental Animal Center of Shandong University for this experiment, and all animal procedures in this study were approved by the Ethics Committee of the Second Hospital of Shandong University (Permit Number: KYLL-2016(GJ)A-0001).

#### Study groups

48 mice were randomly divided into four groups: a wild type group (Sham/WT), RvE1-pretreated wild type group (RvE1), X-ray irradiation group (Rad), and RvE1-pretreated X-ray irradiation group (Rad+RvE1). On days 7 and 14 after irradiation, six mice in each group were sacrificed for experimental analysis.

#### Irradiation method

Experimental mice were irradiated with an Elekta Synergy medical linear accelerator (Elekta, Stockholm, Sweden), 6 MV-X ray, irradiating the inner ear from both sides with the source axis distance (SAD = 100 cm) technique. The irradiation depth of the ear was determined by thin computed tomography (CT) scan with a sample mouse. The irradiation field was 1 cm × 1 cm in size, which fully enveloped the inner ear. The thickness of the lead block was 10 cm, and the radiometric rate was 400 cGy/min.

The irradiation groups were irradiated with 20 Gy in one fraction to the inner ear. This dose was determined according to the results of a preliminary experiment, to ensure robust survival of all experimental mice along with detectable damage to inner ear functions. Before irradiation, 10% chloral hydrate (the Second Hospital of Shandong University, Jinan, China) was used for enterocoelia anesthesia at a dose of 1000 mg/kg without any signs of peritonitis after administration. On the day of the experiments, 1 μg RvE1 (Cayman Chemical, Ann Arbor, USA) was dissolved in 1 mL isotonic saline and administered to the mice via intraperitoneal injection 30 min before irradiation, according to a previous report [11, 16].

### Auditory brainstem response (ABR)

After the experimental mice were anesthetized as described above, they were placed in a soundproof room, the collection electrode was inserted into the top of the skull, the reference electrode was inserted into the mastoid process, and the ground electrode was inserted into the back midline of the mouse. The external speaker was moved to the ear lobe, and clicks and pure tones were performed successively. The selected pure tone frequencies were 4, 8, 16, and 32 kHz, and the results were recorded with a High-frequency Auditory Signal Processor RZ6 Multi-I/O Processor (Tucker Davis Technologies, Alachua, FL, USA) for statistical analysis. ABR tests were performed before irradiation and also on days 7 and 14 after irradiation.

### Roller test

In this test, each mouse was first acclimated to the roller activity for 3 days, ten minutes per day, then on the day of the experiments, the same ten-minute roller activity was performed 30 min before the balance time was officially recorded. The roller speed was 10 r/min during training and testing. During the test, the mice were placed on a rotating roller in turn, and the duration that each mouse stayed on the roller was recorded for three replicates and averaged. Relative balance ability was calculated from the balance time of each mouse on day 7 or day 14 divided by the balance time on the first day before treatment. This test was performed with a Mouse Swivel Fatigue Tester (Biowill Co., Huaibei, China).

### Hematoxylin-eosin (HE) staining

Mice were sacrificed by cervical dislocation, and the cochlea was removed as soon as possible, transferred to precooled 4% PFA and perfused. The cochlea tissue was placed at 4 °C overnight and then washed three times with 1× PBS for 10 min each, followed by 10% EDTA for decalcification. One week later, cochleae were washed with 1× PBS and processed for paraffin embedding. The cochleae were then embedded and sectioned at 4-μm thickness and stained with H&E.

### Whole-mount staining

Cochleae were decalcified in 10% EDTA for 3 days and washed 3 times with 1× PBS for 10 min each. The basal membrane and vestibule were obtained by dissecting the cochleae and then permeabilized with 0.2% Trition X-100 for 8 min. The basal membrane and vestibule were then blocked for 30 min with 10% normal goat serum (NGS). The Myosin VIIa (myo7a) antibody (Abcam, Cambridge, MA, USA), diluted 1:200, was added to the basal membrane and vestibule and left overnight at 4 °C. The membrane and vestibule were then washed and incubated with 1:200 mouse-IgG 594 (Bethyl, Montgomery, AL, USA) for 1 h and then with 1:500 phalloidin (Sigma Aldrich, St Louis, MO, USA) for 40 min. After washing with 1× PBS 3 times, DAPI (Sigma Aldrich) at a 1:250 dilution was added and incubated for 10 min at room temperature. The specimens were sealed by glycerin, and organs of Corti, spiral ganglion, utricle and sacculus were imaged using a confocal microscope (LSM700; Carl Zeiss, Germany).

### Scanning Electron microscopy (SEM)

Cochleae were decalcified in 10% EDTA for 3 days and then soaked in precooled 2.5% glutaraldehyde (Beijing Dingguo Changsheng Biotechnology Co. LTD, Beijing, China). Cochleae were dissected to obtain the basal membrane and vestibule, which were washed three times with 1× PBS for 10 min each. Then, osmium tetroxide was added in a fume hood. Samples were washed again three times with 1× PBS and 4 times with sterile distilled water, before dehydrating through an ethanol gradient. Then, the critical point was selected for drying the sample with carbon dioxide for 2 h. Dried specimens were sputter coated with gold for 4 min. Following the preparation process, the samples were observed under a scanning electron microscope (Zeiss sigma 300, Carl Zeiss, Germany).

### Quantitative real-time polymerase chain reaction (qRT-PCR)

At the indicated days, mice were sacrificed for dissection of the basal membrane, modiolus, and vestibule. RNA was extracted from each tissue using TRIzol reagent (Invitrogen, Carlsbad, CA, USA) according to the manufacturer’s protocol. To ensure that total RNAs met the requirements for the experiment, the purity and concentration of RNA were quantified using the Nano Drop ND-1000 Spectrophotometer (Thermo Fisher Scientific, Waltham, MA, USA), and the integrity of RNA was assessed with the Agilent 2100 Bioanalyzer (Agilent Technologies, Santa Clara, CA, USA). Total cDNA was synthesized using a one-step cDNA synthesis kit (Vazyme, Nanjing, China). Using cDNA as a template, changes in the expression of inflammatory factors (IL-2, IL-6, IL-10, tumor necrosis factor [TNF]-α, and interferon [IFN]-γ) in the tissues were analyzed by qRT-PCR. The qRT-PCR reactions were carried out using a 7900 HT Fast RealTime PCR system (Applied Biosystems, Foster City, CA, USA) and the reaction conditions were as follows: pre-denaturation at 95 °C for 5 min; denaturation at 95 °C for 30 s, annealing at 58 °C for 30 s, extension at 68 °C for 25 s, 40 cycles; 72 °C extension for 5 min, and hold at 4 °C. Relative gene expression was calculated using the comparative 2^-△△Ct^ method with the housekeeping gene mus-β-actin as a reference. All primers were synthesized by Invitrogen and sequences were as follows: IL-2 forward primer: 5′-AGATGAACTTGGACCTCTGCG-3′, reverse primer: 5′-AAAGTCCACCACAGTTGCTG-3′ (175 bp). IL-6 forward primer: 5′-TTCCTCTGGTCTTCTGGAGT-3′, reverse primer: 5′-GAGAGCATTGGAAATTGGGGT-3′ (181 bp). IL-10 forward primer: 5′-AGCCTTATCGGAAATGATCCAGT-3′, reverse primer: 5′-GGCCTTGTAGACACCTTGGT-3′ (229 bp). TNF-α forward primer: 5′-CCTGTAGCCCACGTCGTAG-3′, reverse primer: 5′-GGGAGTAGACAAGGTACAACCC-3′ (148 bp). IFN-γ forward primer: 5′-ACAGCAAGGCGAAAAAGGATG-3′, reverse primer: 5′-TGGTGGACCACTCGGATGA-3′ (106 bp). β-actin forward primer: 5′-ACGGCCAGGTCATCACTATTG-3′, reverse primer: 5′-AGGGGCCGGACTCATCGTA-3′ (372 bp).

### Statistical analysis

Statistical analysis was performed using SPSS 20.0 software (IBM SPSS Statistics for Windows, Armonk, NY, USA). The expression levels of inflammatory factors, hearing threshold and relative balance ability were expressed as the mean ± standard deviation (SD) and the differences among different groups were compared by one-way analysis of variance (ANOVA), wihle multiple comparisons among different subgroups were tested using the Bonferroni test. All *P*-values were two-sided, and a value of *P* < 0.05 was considered statistically significant.

## Results

### RvE1 alone has no effects on the inner ear

**Fig. 1:**
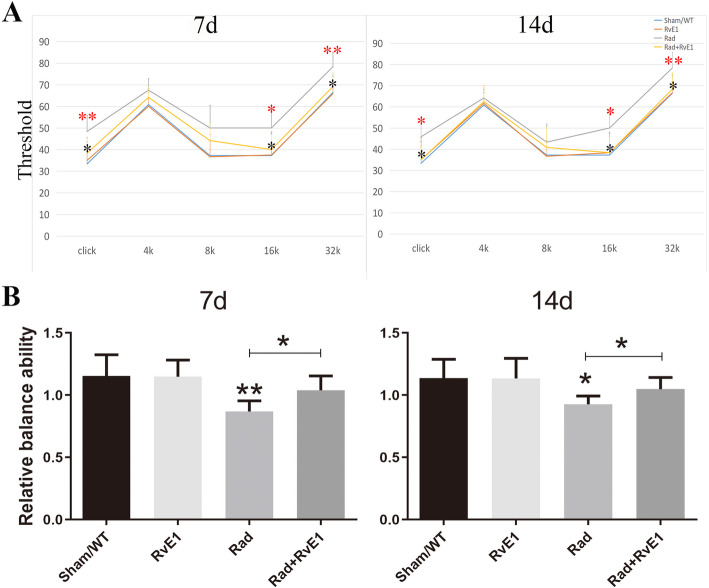
One-way analysis of variance for hearing threshold and relative balance ability showed the changes among the different groups after irradiation and resolvin E1-pretreatment. ^*^, *P* ≤ 0.05; ^**^, *P* ≤ 0.01; **a**. Hearing changes after irradiation and resolvin E1-pretreatment. Irradiation-induced hearing loss, which was obvious on days 7 and 14 after irradiation; resolvin E1 (RvE1) mitigated the irradiation-induced hearing loss. The red asterisk indicates a comparison between the Sham/WT and Rad groups. The black asterisk indicates a comparison between the Rad and Rad+RvE1 groups. **b**. Relative balance ability changes after irradiation and resolvin E1-pretreatment. The relative balance ability in Sham/WT, RvE1, Rad and Rad+RvE1 groups were respectively 1.154 ± 0.171, 1.149 ± 0.133, 0.869 ± 0.085, and 1.039 ± 0.115 on day 7 and 1.137 ± 0.151, 1.134 ± 0.161, 0.927 ± 0.066, and 1.048 ± 0.094 on day 14. Irradiation induced damage in relative balance ability (compared between the Sham/WT and Rad groups) on days 7 and 14 after irradiation (*P* = 0.0045 and *P* = 0.0107), and RvE1 mitigated this damage (comparison between the Rad and Rad+RvE1 groups, *P* = 0.0155 on day 7 and *P* = 0,0270 on day 14)

**Fig. 2:**
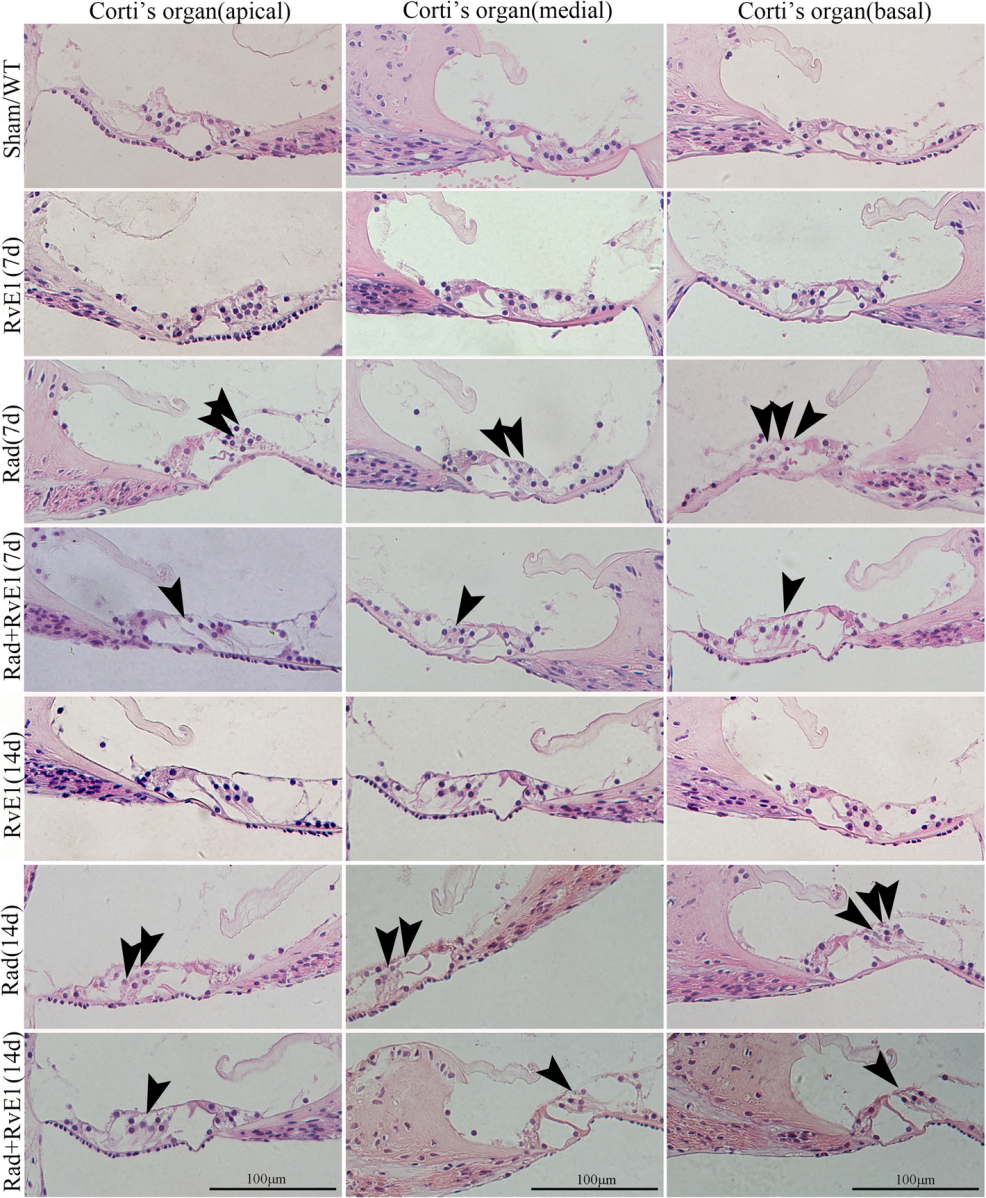
Hematoxylin-eosin staining showed the changes to the hair cells of the basement membrane after irradiation and resolvin E1-pretreatment. The arrows indicate the points of the loss of basement membrane hair cells. The loss could be observed in the apical, medial and basal gyrus of the cochlea on days 7 and 14 after irradiation. Pretreatment with resolvin E1 (RvE1) clearly reduced this damage. An obvious reduction in the number of hair cells can be observed at the points indicated by the arrows in the Rad+RvE1 group compared to the Rad group

**Fig. 3:**
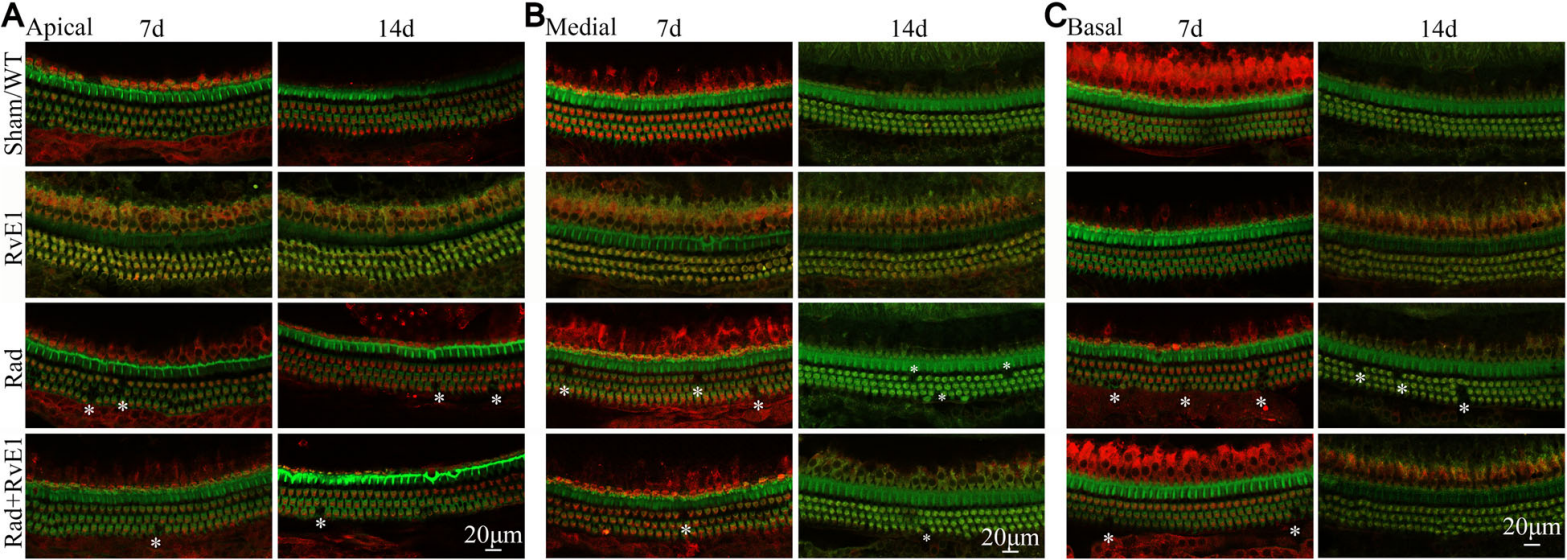
Whole-mount staining showed changes to the hair cells of the basement membrane after irradiation and resolvin E1-pretreatment. The asterisks indicate the points of the loss of hair cells, and the number of asterisks represents the frequency of damage. The loss was observed on days 7 and 14 after irradiation, but this damage was markedly reduced by pretreatment with resolvin E1

**Fig. 4:**
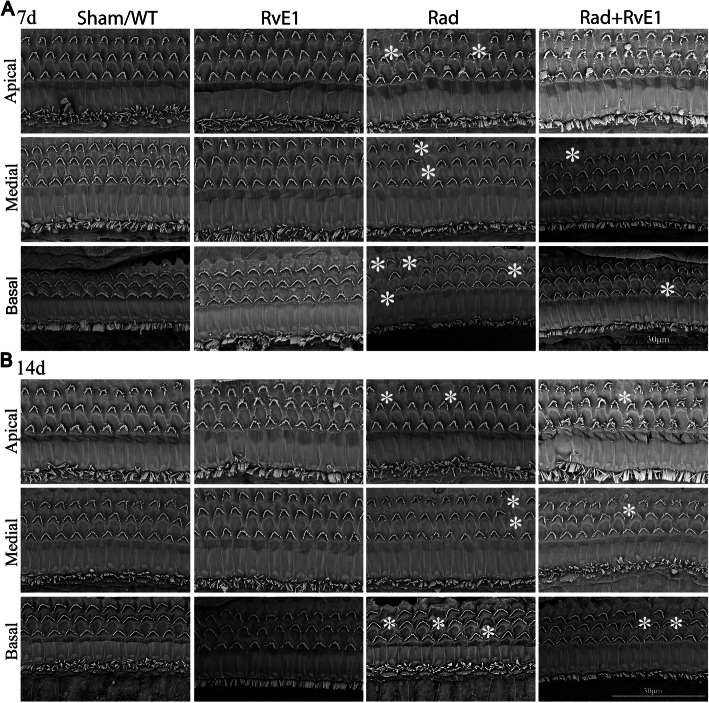
Scanning electron microscopy showed changes to the hair cells of the basement membrane after irradiation and resolvin E1-pretreatment. The asterisks indicate the points of the loss of hair cells, and the number of asterisks represents the frequency of damage, which could be observed on days 7 and 14 after irradiation. However, pretreatment with resolvin E1 obviously reduced this damage

**Fig. 5:**
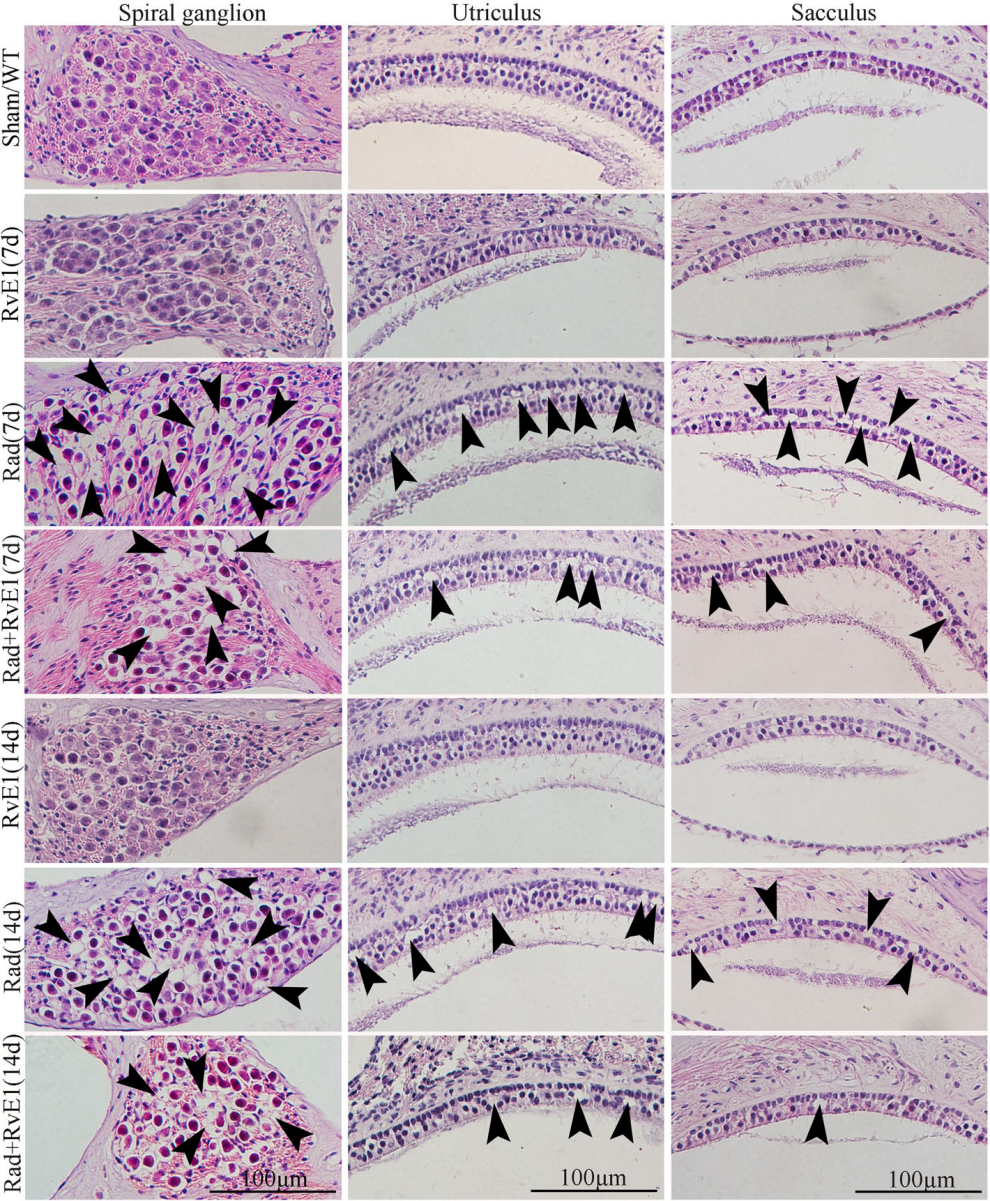
Hematoxylin-eosin staining showed changes to the spiral ganglion cells and hair cells of the utriculus and sacculus after irradiation and resolvin E1-pretreatment. The arrows indicate the points of the loss of spiral ganglion cells and utriculus and sacculus hair cells, and the number of arrows represents the frequency of damage, which could be observed on days 7 and 14 after irradiation. Meanwhile, pretreatment with resolvin E1 mitigated the damage. The loss of utriculus and sacculus hair cells represents that of vestibular hair cells

**Fig. 6:**
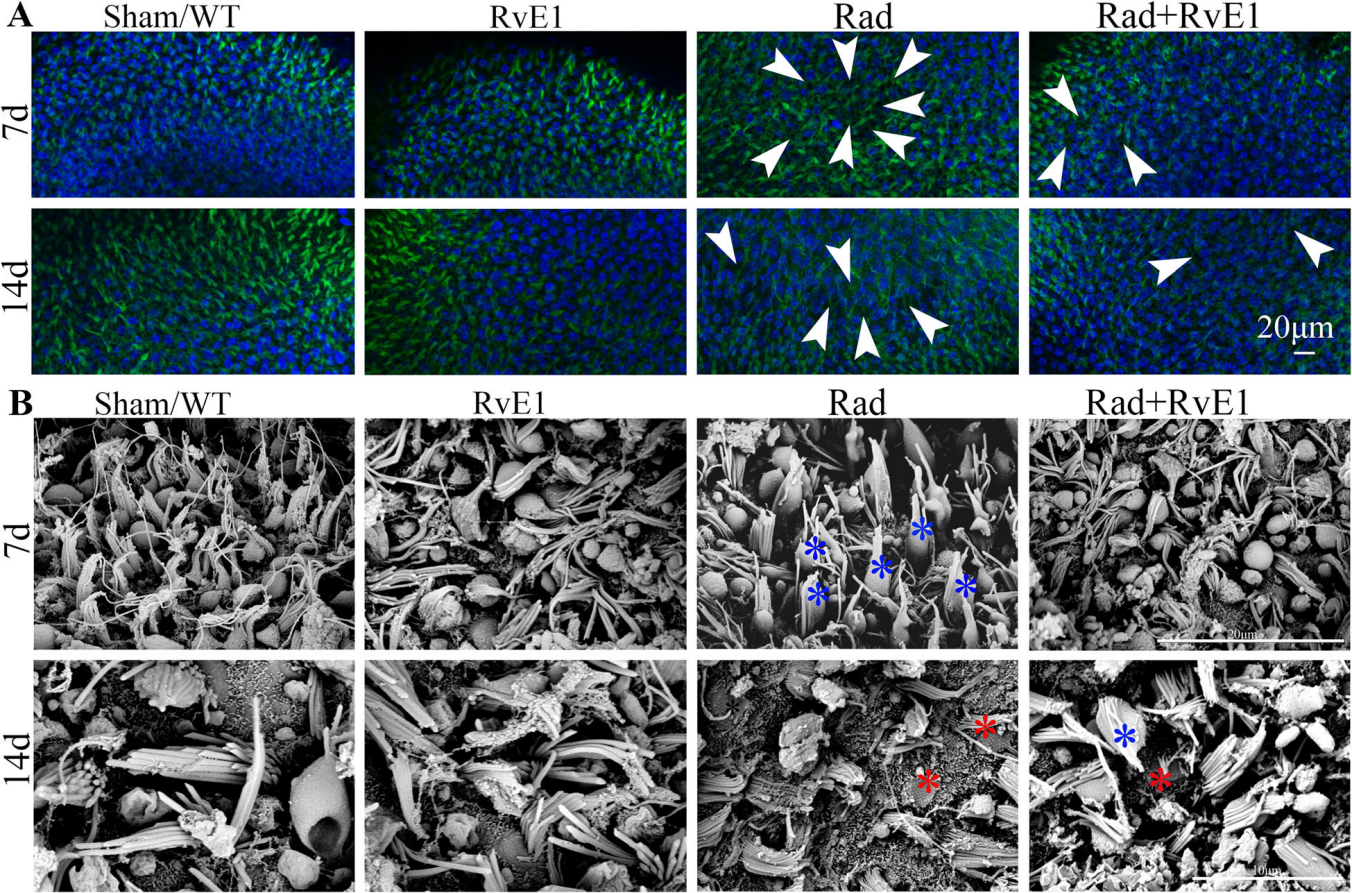
Whole-mount staining (**a**) and scanning electron microscopy (**b**) showed changes to the vestibular hair cells after irradiation and resolvin E1-pretreatment. The arrows (**a**) indicate the points of the loss of vestibular hair cells. The red asterisks (**b**) indicate the points of cilia fusion and blue asterisks (**b**) indicate the points of cilia loss of vestibular hair cells, while the numbers of arrows and asterisks represent the frequencies of damage, which could be observed on days 7 and 14 after irradiation. Pretreatment with resolvin E1 obviously reduced this damage of cells

**Fig. 7:**
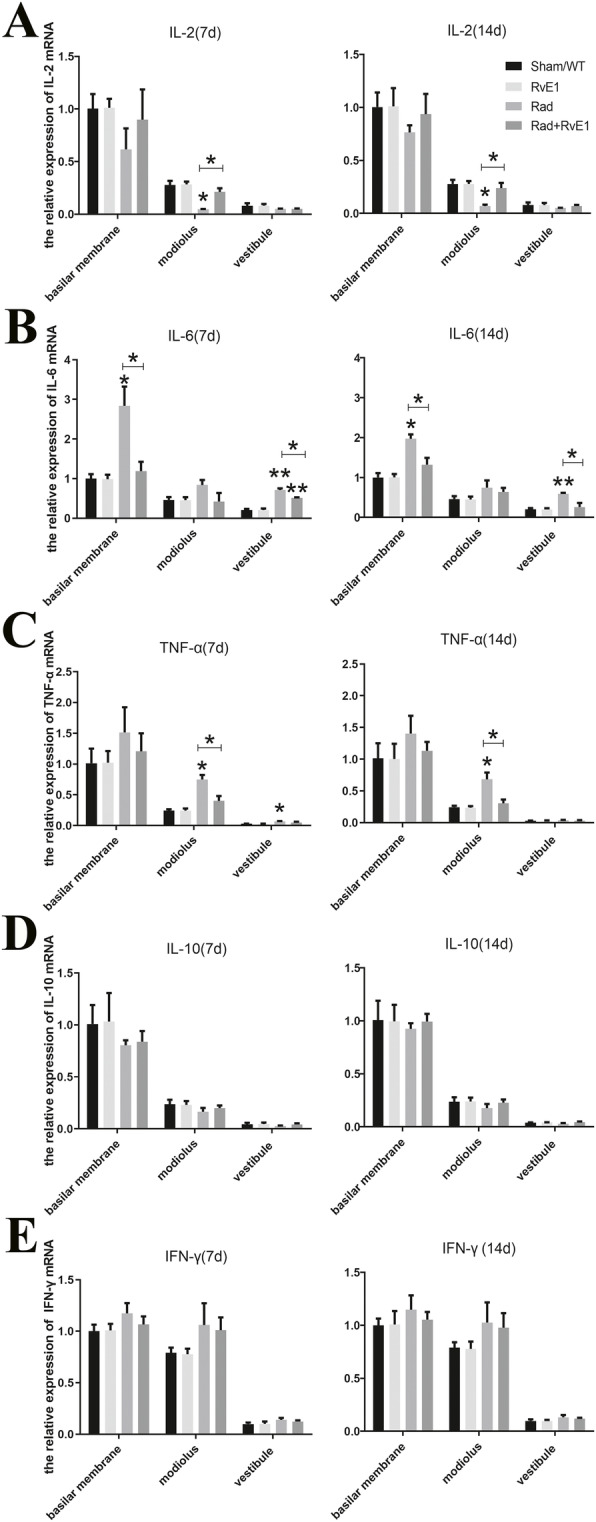
One-way analysis of variance for inflammatory factor expression showed the changes among the different groups after irradiation and resolvin E1-pretreatment. ^*^, *P* ≤ 0.05; ^**^, *P* ≤ 0.01; **a**. The expression level changes of IL-2 among the different groups. The expression level of IL-2 in the modiolus was significantly decreased on days 7 and 14 after irradiation (compared between the Sham/WT and Rad groups). **b**. The expression level changes of IL-6 among the different groups. The expression levels of IL-6 in the basal membrane and vestibule were significantly increased on days 7 and 14 after irradiation (compared between the Sham/WT and Rad groups). **c**. The expression level changes of TNF-α among the different groups. The expression levels of TNF-α in the modiolus and vestibule were significantly increased on day 7 after irradiation (compared between the Sham/WT and Rad groups), and a similar significant change was observed only in the modiolus on day 14 after irradiation (compared between the Sham/WT and Rad groups). **d** and **e**. The expression level changes of IL-10 and IFN-γ among the different groups. The expression of IL-10 and IFN-γ did not significantly change among the different groups. All these significant changes after irradiation could be ameliorated by resolvin E1 (RvE1) pretreatment (compared between the Rad and Rad+RvE1 groups)

Six mice from each of the Sham/WT, RvE1, Rad and Rad+RvE1 groups were selected for ABR and roller tests, morphological analysis and the detections of inflammatory factors on days 7 and 14 of the experiments. No differences were observed between Sham/WT and RvE1 groups in the functions, morphology or the levels of inflammatory factors in the inner ear of experimental mice at either of the two time points (Figs. 1, 2, 3, 4, 5, 6 and 7). The subgroup comparisons of Sham/WT group with Rad or Rad+RvE1 groups and those of RvE1 group with Rad or Rad+RvE1 groups showed the same results, which confirmed that RvE1 alone has no effects on the inner ear. Therefore, the compared data among RvE1, Rad and Rad+RvE1 groups are not described repeatedly in the following sections in order to simplify the figures and avoid confusing description while comparing among different groups.

### RvE1 protects the inner ear function after irradiation

During experiments, ABR and roller tests were used to evaluate changes in hearing and relative balance ability in the inner ear.

During the ABR test, each group was tested using clicks and pure tones at 4, 8, 16, and 32 kHz. On days 7 and 14 after irradiation, there were significant differences in the pure tone thresholds of 16 kHz and 32 kHz and in the click tone between the Sham/WT and the Rad groups and between the Rad and Rad+RvE1 groups (Fig. 1a). The results also showed that RvE1 pretreatment obviously ameliorated this damage. There was no significant difference among the different groups at 4 or 8 kHz (Fig. 1a). These results implied that RvE1 had a beneficial radioprotective effect on the hearing of the mice, and the radioprotective effect acted primarily on the medial and basal gyrus rather than the apical gyrus of the cochlea.

The results of the roller test showed that the relative balance ability of mice in the Rad group had decreased significantly by days 7 and 14 compared to that in the Sham/WT group (Fig. 1b). However, after RvE1 pretreatment, the relative balance ability of mice in the Rad+RvE1 group was improved significantly compared to that in the Rad group, while no differences were evident between the Sham/WT and Rad+RvE1 groups (Fig. 1b). These results confirmed that the balance function of the inner ear was damaged by irradiation, and that RvE1 could mitigate the damage to the inner ear. At the same time, these also implied that the radioprotective effect of RvE1 acted primarily on the vestibule.

### RvE1 has protective effects on irradiated basement membrane hair cells

Cochlear tissues of each group were collected on days 7 and 14 after irradiation. Hair cells were labeled with Myo7a, and phalloidin was used to label hair cell actin. Morphological changes of the basement membrane hair cells in the cochlea were observed by HE staining, whole-mount staining and SEM. HE staining (Fig. 2) and whole-mount staining (Fig. 3) showed obvious loss of basement membrane hair cells in the apical, medial and basal gyrus of the cochlea on days 7 and 14 after irradiation. The points of the loss are indicated by arrows in Fig. 2 and by asterisks in Fig. 3. SEM (Fig. 4) also showed obvious loss of the cilia of basement membrane hair cells by days 7 and 14 after irradiation; the asterisks indicate the points of the loss. RvE1 pretreatment did not eliminate the damage, but the degree of damage was substantially reduced compared to irradiation alone. Therefore, we believe that RvE1 exerts radioprotective effects on basement membrane hair cells.

### RvE1 has a protective effect on spiral ganglion cells after irradiation

The modiolus of each group was harvested for analysis on days 7 and 14 after irradiation. We observed morphological changes of spiral ganglion cells by HE staining (Fig. 5). HE staining showed that the loss of spiral ganglion cells could be obviously observed on days 7 and 14 after irradiation; the points of the loss are indicated with arrows in Fig. 5. Pretreatment with RvE1 did not eliminate the damage, but the degree of damage was reduced compared to irradiation alone. Therefore, we believe that RvE1 exerted a radioprotective effect on spiral ganglion cells.

### RvE1 has protective effects on vestibular hair cells after irradiation

The vestibular tissues of each group were harvested on days 7 and 14 after irradiation. The vestibular hair cells were labeled with DAPI, and vestibular hair cell actin was labeled with phalloidin. Morphological changes of vestibular hair cells were observed by HE staining, whole-mount staining and SEM. HE staining (Fig. 5) and whole-mount staining (Fig. 6a) showed there was some loss of vestibular hair cells on days 7 and 14 after irradiation. Using SEM (Fig. 6b), we also observed cilia fusion (red asterisks) and loss (blue asterisks) in vestibular hair cells on days 7 and 14 after irradiation. All the points of damage are indicated by black arrows in Fig. 5, white arrows in Fig. 6a and red and blue asterisks in Fig. 6b. The loss of utriculus and sacculus hair cells in Fig. 5 represented that of vestibular hair cells. After RvE1 pretreated, the degree of damage was obviously reduced compared to irradiation alone. This indicated that RvE1 also had a radioprotective effect on vestibular hair cells.

### RvE1 alleviates inflammation of the inner ear caused by irradiation

To confirm that the inflammatory response induced by irradiation was involved in the damage to the inner ear after radiotherapy, changes in inflammatory factor expression in the inner ear of mice after irradiation were analyzed by qRT-PCR. The results showed that the expression level of IL-2 was significantly decreased only in the modiolus on days 7 and 14 after irradiation, while after pretreatment with RvE1, the IL-2 level in the modiolus was obviously increased compared to irradiation alone (Fig. 7a). The expression levels of IL-6 in the basilar membrane and vestibule were significantly increased on days 7 and 14 after irradiation, but after pretreatment with RvE1, IL-6 levels in the basilar membrane and vestibule were obviously decreased compared to the group that received irradiation alone. Although the IL-6 level in the modiolus also changed similarly, there were no significant differences among the different groups (Fig. 7b). The expression level of TNF-α in the modiolus was also significantly increased on days 7 and 14 after irradiation, and again, after pretreatment with RvE1, the TNF-α level in the modiolus was obviously reduced compared to the group that received irradiation alone. A similar significant change was also observed in the vestibule on day 7. No other changes were significant among the different groups (Fig. 7c). The expression levels of IL-10 and IFN-γ did not show any significant changes on day 7 or 14 after irradiation among the different groups (Fig. 7d and f). These results implied that RvE1 could cause expression level changes of some inflammatory factors in inner ear, which together might be involve in irradiation damage to the inner ear. The results also suggested that RvE1 might protect the inner ear from irradiation damage by regulating the inflammatory response induced by irradiation, which was associated with these inflammatory factors.

## Discussion

The inner ear is an important organ at risk (OAR) in patients with head and neck cancers receiving radiotherapy, and SNHL and vestibular dysfunction are often inevitable. Some studies suggest that the incidence of post-irradiation SNHL may be as high as 43% [20]. The main cause of irradiation-induced SNHL is thought to be the direct action of irradiation on cochlear hair cells and the spiral ganglion, leading to damage to cell and mitochondrial membranes, lysosomal membrane lipid peroxidation and DNA damage and subsequently resulting in cellular metabolic disorder, hair cell degeneration, necrosis, and dysfunction [21, 22]. Damage to the vasculature can cause endolymphatic edema, which can also lead to hearing loss, tinnitus, ear distension, vertigo or balance disorders [22, 23]. In this study, we also observed hearing loss in mice, evaluated by ABR, along with HE staining, whole-mount staining and SEM showing various degrees of damage to the cochlea basal membrane hair cells and spiral ganglion cells after irradiation. Post-irradiation vestibular dysfunction has rarely been reported, and our previous study found an incidence of post-irradiation vestibular dysfunction of up to 60–70% in clinical practice [9], but the mechanism has not previously been reported. In this study, post-irradiation vestibular dysfunction in mice was also indicated by the results of relative balance ability tests, and HE staining, whole-mount staining and SEM all showed that damage to utriculus and sacculus hair cells after irradiation was the main cause of post-irradiation vestibular dysfunction.

The direct damaging effect of irradiation on the target organ is the main cause of post-irradiation inner ear damage, but another cause of post-irradiation inner ear damage is the irradiation-induced inflammatory response. Some studies have reported that various inflammatory factors significantly increased after radiotherapy, mainly proinflammatory cytokines such as IL-1, IL-6, IL-8, and TNF-α [24]. Our previous study found that IL-6 was significantly increased in nasopharyngeal carcinoma patients after radiotherapy and was associated with post-irradiation vestibular dysfunction [9]. These results supported the hypothesis that the irradiation-induced inflammatory response participated in vestibular dysfunction. In this study, we directly observed a significant decrease in the mRNA level of IL-2 and significant increases in the mRNA levels of IL-6 and TNF-α in mouse inner ears at different time-points after irradiation. These results confirmed the potential involvement of the inflammatory response associated with these inflammatory factors in post-irradiation inner ear damage.

RvE1 is a trihydroxy derivative of the polyunsaturated fatty acid EPA, and is found in fish oil and sea food. RvE1 possesses a remarkable treatment effect for many inflammation-related diseases and conditions when administered via subcutaneous injection, intraperitoneal injection, intraplantar injection and intrathecal injection [11, 13–17, 25]. In this study, after RvE1 was administered via intraperitoneal injection, we observed amelioration of inner ear damage after irradiation and changes of some of the inflammatory factors associated the inflammatory response.

There have been no previous reports regarding whether RvE1 has a radioprotective effect on the inner ear. However, based on the fact that a local inflammatory response in irradiated tissues is a universal occurrence, and further, that RvE1 has remarkable effects in counteracting inflammation and promoting resolution of inflammation, we explored the radioprotective effect of RvE1 on the inner ear via experiments in a mouse model. As a result, we found that RvE1 considerably ameliorated irradiation-related damage to the inner ear. In this study, employing RvE1 pretreatment before irradiation, we not only observed the alleviation of hearing loss and vestibular dysfunction, but also observed the amelioration of microstructure damage associated with hearing loss and vestibular dysfunction. These results proved that the local inflammatory response in irradiated tissues was involved in tissue damage, and at the same time, also proved that RvE1 had a radioprotective effect on the inner ear.

The molecular mechanisms underlying the anti-inflammatory activity of RvE1 and its promotion of inflammation resolution are complicated [15, 17, 18, 26]. Among these complicated mechanisms, RvE1 modulation of inflammation-related cytokine expression plays an important role. Therefore, in this study, we investigated changes in the expression of the inflammatory factors IL-2, IL-6, IL-10, TNF-α, and IFN-γ after RvE1 pretreatment to explore the potential mechanisms via which RvE1 protects against irradiation-induced damage to the inner ear. Among these inflammatory factors, IL-2 and IL-10 are anti-inflammatory factors, which can inhibit the inflammatory cascade reaction by downregulating the expression of proinflammatory factors [19, 27]. IL-6, TNF-α, and IFN-γ are important proinflammatory factors that activate the inflammatory cascade reaction and cause damage to target organs [19, 27]. The influence of RvE1 on these inflammatory factors has been previously reported. Xu et al. reported that RvE1 ameliorated pulpitis by inhibiting the expression of the proinflammatory cytokines IL-6 and TNF-α in a chemerin receptor23 (ChemR23)-dependent manner [28]. Rey et al. reported that RvE1 decreased LPS-induced gene expression of proinflammatory cytokines (TNF-α and IL-6), suggesting that they exerted proresolutive activity in microglia [29]. Wang et al. reported that RvE1 treatment improved allogeneic corneal graft survival by significantly reducing the mRNA expression of proinflammatory cytokines, including TNF-α, IL-6 and IFN-γ, in corneal grafts, as well as the protein level of the proinflammatory cytokines TNF-α, IL-6 and IFN-γ [30]. However no similar findings have been reported in the inner ear.

In this study, the mRNA expression level of IL-2 only increased significantly in the modiolus at different time-points after RvE1 pretreatment compared to irradiation alone. In contrast, the mRNA expression levels of IL-6 and TNF-α decreased significantly in more extensive parts of the inner ear after irradiation along with RvE1 pretreatment. Further analysis found that the changes in expression of IL-2, IL-6, and TNF-α were also associated with damage to cochlear hair cells, spiral ganglion cells, and vestibular sacculus and utriculus hair cells. All of these results implied that changes in the expression levels of these inflammatory factors in the inner ear together affected the damage caused to the inner ear after irradiation, therefore the inflammatory response induced by irradiation might be involved in the damage, and some proinflammatory cytokines might play more important roles in this response. Further, RvE1 was able to ameliorate this kind of damage to the inner ear, and its radioprotective mechanism might be mediated through its anti-inflammatory effects via regulation of IL-2, IL-6, and TNF-α.

Regarding the way in which RvE1 regulates the expressions of IL-2, IL-6, and TNF-α, current consensus is that RvE1 mediates its effects via binding to the ChemR23 and leukotriene B4 receptor1 (BLT1). In particular, ChemR23 is widely expressed on monocytes, macrophages, dendritic cells and epithelial cells [31, 32] and is upregulated in response to tissue damage or inflammatory stimuli [33, 34]. Within the inflammatory microenvironment induced by irradiation, these cells are universally present, and mediated the release of numerous inflammatory factors and inflammatory damage to tissue [19]. However, in this study it was unclear whether RvE1 regulated the expressions of IL-2, IL-6, and TNF-α in the inner ear via such ChemR23 and BLT1 receptor-dependent mechanisms. This question is worthy of study in the future.

## Conclusions

In summary, post-irradiation damage to the cochlear hair cells, spiral ganglion cells, and vestibular sacculus and utriculus hair cells led to SNHL and vestibular dysfunction. The inflammation induced by irradiation participated in the functional damage to the inner ear following irradiation. RvE1 protected the inner ear of mice from post-irradiation damage through its anti-inflammatory effects, which might be mediated by regulation of IL-2, IL-6, and TNF-α expression.

## Data Availability

The materials used and/or analyzed in the current study are available from the corresponding author on reasonable request.

## Abbreviations

SNHL: Sensorineural hearing loss
IL-6: Interleukin-6
RvE1: Resolvin E1
EPA: Eicosapentaenoic acid
WT: Wild type group
Rad: X-ray irradiation group
Rad+RvE1: RvE1-pretreated X-ray irradiation group
SAD: Source axis distance
CT: Computed tomography
ABR: Auditory brainstem response
HE: Hematoxylin-eosin
SEM: Scanning electron microscopy
myo7a: Myosin VIIa
qRT-PCR: Quantitative real-time polymerase chain reaction
TNF-α: Tumor necrosis factor-α
IFN-γ: Interferon-γ
IL-2: Interleukin-2
IL-10: Interleukin-10
SD: Standard deviation
ANOVA: One-way analysis of variance
OAR: Organ at risk
ChemR23: Chemerin receptor23
BLT1: Leukotriene B4 receptor1
